# Filtration Device for On-Site Collection, Storage and Shipment of Cells from Urine and Its Application to DNA-Based Detection of Bladder Cancer

**DOI:** 10.1371/journal.pone.0131889

**Published:** 2015-07-07

**Authors:** Elin Andersson, Christina M. Dahmcke, Kenneth Steven, Louise K. Larsen, Per Guldberg

**Affiliations:** 1 Danish Cancer Society Research Center, Copenhagen, Denmark; 2 Department of Pathology, Copenhagen University Hospital, Herlev, Denmark; 3 Department of Urology, Copenhagen University Hospital, Herlev, Denmark; CEA—Institut de Genomique, FRANCE

## Abstract

Molecular analysis of cells from urine provides a convenient approach to non-invasive detection of bladder cancer. The practical use of urinary cell-based tests is often hampered by difficulties in handling and analyzing large sample volumes, the need for rapid sample processing to avoid degradation of cellular content, and low sensitivity due to a high background of normal cells. We present a filtration device, designed for home or point-of-care use, which enables collection, storage and shipment of urinary cells. A special feature of this device is a removable cartridge housing a membrane filter, which after filtration of urine can be transferred to a storage unit containing an appropriate preserving solution. In spiking experiments, the use of this device provided efficient recovery of bladder cancer cells with elimination of >99% of excess smaller-sized cells. The performance of the device was further evaluated by DNA-based analysis of urinary cells collected from 57 patients subjected to transurethral resection following flexible cystoscopy indicating the presence of a tumor. All samples were tested for *FGFR3* mutations and seven DNA methylation markers (*BCL2*, *CCNA1*, *EOMES*, *HOXA9*, *POU4F2*, *SALL3* and *VIM*). In the group of patients where a transitional cell tumor was confirmed at histopathological evaluation, urine DNA was positive for one or more markers in 29 out of 31 cases (94%), including 19 with *FGFR3* mutation (61%). In the group of patients with benign histopathology, urine DNA was positive for methylation markers in 13 out of 26 cases (50%). Only one patient in this group was positive for a *FGFR3* mutation. This patient had a stage Ta tumor resected 6 months later. The ability to easily collect, store and ship diagnostic cells from urine using the presented device may facilitate non-invasive testing for bladder cancer.

## Introduction

Analysis of rare cells present in complex biological fluid samples provides a potentially powerful diagnostic and assessment tool for a range of diseases and conditions. In particular, isolation, quantitation and downstream testing of cancerous cells present in patient body fluid samples hold great promise for non-invasive detection and characterization of tumors to guide diagnostic and therapeutic decisions. A common approach to cancer diagnostics through minimally invasive sampling is by isolation of intact tumor cells or cell-free tumor DNA from peripheral blood samples [1,2]. The ability to analyze circulating tumor-derived material has been rapidly advanced by major technological developments and the discovery of highly informative biomarkers, including some that represent targets for precision cancer therapies [3].

For urological cancers such as bladder cancer, urine provides a more convenient source of diagnostic material. Cells shed from tumors located in the urinary tract accumulate in the bladder and can be collected and analyzed non-invasively by urine sampling [4]. Urine cytology has been widely used to diagnose urological cancers, particularly as an adjunct to cystoscopy for detection and surveillance of bladder cancer. However, for low-grade bladder tumors, cytology has a sensitivity as low as 10–20% [5] and has been abandoned by many centers. Several urinary tests for bladder cancer have been approved by the US Food and Drug Administration (FDA), but their performance is still inferior to cystoscopy in terms of sensitivity (true positive rate) and specificity (true negative rate) [6]. Greater performance may be achieved by using gene-based urinary biomarkers such as driver mutations and DNA methylation alterations, which are cancer specific and less affected by inflammation and other benign conditions [7–12]. With the advent of improved methods for detection and quantitation of rare DNA molecules, including next-generation sequencing and digital PCR [13], the sensitivity of DNA-based detection of bladder tumors may be further increased.

Despite its promise, the use of urinary cell-based assays for detection of bladder cancer is limited by inherent challenges of collecting and processing urine specimens. The most common procedure for analyzing the cellular content of urine involves sedimentation of cells by centrifugation. To avoid cell lysis and degradation of cellular components, samples should be processed quickly after voiding. For these practical reasons, sampling is usually performed at a dedicated site with specialized equipment and trained personnel. Another important aspect related to the performance of urine-based tests is the high intra- and inter-individual variation in total urinary cell count and ratio of tumor-to-normal cells [14]. A high background of normal cells limits the sensitivity of most detection assays and requires that a larger fraction of the sample material be analyzed to increase the chance of identifying tumor cells.

The cellular component of urine is highly heterogeneous, consisting of cells of various types and sizes, such as epithelial cells, squamous cells and macrophages [15,16]. We [17] and others [18–20] have previously shown that pre-analytic filtration of urine using a membrane filter provides a means for capturing and enriching bladder cancer cells from urine. With a pore size of approximately 8 μm, such filters can capture normal and malignant urothelial cells, which typically have a diameter of >20 μm, while they eliminate some smaller-sized cells. A typical procedure for filtration, such as the one used in our previous study [17], involves the use of a disposable syringe to force urine through a membrane filter mounted in an appropriate filter holder equipped with an inlet and an outlet. After filtration, the filter is removed with tweezers and transferred to a micro-centrifuge tube for immediate processing or freezer storage. This procedure requires training, is not suited for processing large volumes and implies the risk of losing material through spillage and during handling of the delicate filter membranes.

We present a filtration device for sampling and storage of cells from large volumes of urine or other biological fluids. After filtration, the filter with captured cells can be readily removed from the filtration device and placed into a water-tight storage unit, which contains an appropriate solution for preparing or preserving the captured cells. The storage unit can then be shipped to a testing facility for appropriate downstream analysis of the cellular content. We demonstrate the use of this device for DNA-based characterization of patients with benign lesions and malignant tumors of the bladder.

## Materials and Methods

### Patients and urine samples

Urine samples were collected from patients in whom routine flexible cystoscopy in the outpatient clinic showed evidence of bladder tumor and who were subsequently referred to transurethral resection of the bladder (TURB) at Copenhagen University Hospital, Herlev, Denmark. The study included both newly diagnosed patients and patients undergoing routine check cystoscopy as follow-up of previously diagnosed transitional cell tumor of the bladder. Samples were transported at room temperature to the Danish Cancer Society and processed within 4–6 hours after voiding. The study was approved by The Committee on Biomedical Research Ethics of the Capital Region of Denmark (H-2-2010-045), and written informed consent was obtained from all subjects before study participation.

### Collection of cells and DNA isolation

**Fig 1:**
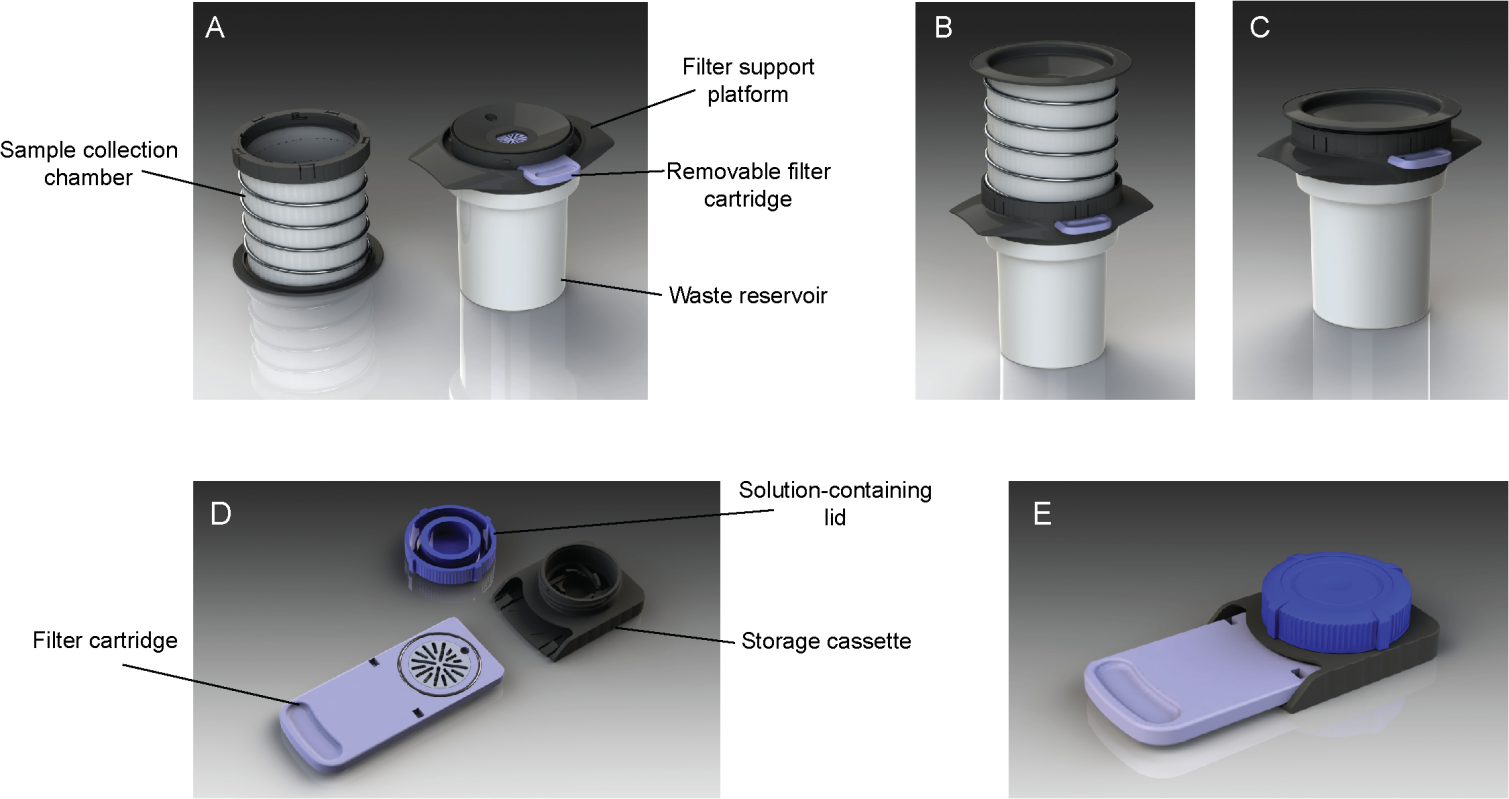
Outline of the filtration device and its components. The device is composed of a filtration unit (A, individual components; B, assembled device; C, after filtration) and a water-tight storage cassette (D, individual components; E, assembled cassette).

Voided urine samples (100–400 ml) or suspensions of cultured cells were processed using a filtration device (Fig 1) mounted with a Nuclepore track-etched polycarbonate hydrophilic membrane filter (diameter 25 mm, pore size 8 μm; Whatman, Maidstone, UK). After filtration, the filter cartridge was transferred to the storage cassette, which was then mounted with the lid from an Oragene DNA Self-Collection Kit containing 1.7 ml of lysis/stabilization buffer (disc format OG-250; DNA Genotek, Ottowa, Ontario, Canada). According to the manufacturer, DNA is stable in this solution for >5 years at room temperature. DNA was purified from 0.5 ml of sample using the Oragene DNA purifying solution (DNA Genotek), according to the ethanol precipitation protocol provided by the manufacturer. For collection of the total cellular component, 25 ml of urine were centrifuged at 2,000 g for 10 min. The pellet was resuspended in 200 μl of phosphate-buffered saline (PBS) and stored at -80°C. DNA was extracted using the Qiagen Mini Prep kit (Qiagen GmbH, Hilden, Germany) according to the manufacturer’s instructions (protocol for DNA purification from tissues). DNA yield was estimated by quantitative real-time PCR analysis, using a LightCycler 2.0 system (Roche, Mannheim, Germany), the FastStart DNA Master^PLUS^ SYBR Green I Kit (Roche), Human Genomic DNA (Roche) as reference and the following primers for *GAPDH*: 5’-TAGTGTCCTGCTGCCCACAGTCCAG-3’ and 5’-GGCGACGCAAAAGAAGATGC-3’.

### Cell culture and model system

The urothelial carcinoma-derived cell lines 639V and 97–7 were from the laboratory of Margaret A. Knowles and harbor the *FGFR3* p.R248C and p.S249C mutations, respectively [21]. Cells were maintained in DMEM medium supplemented with 10% fetal bovine serum at 37°C in a humidified incubator with 5% CO_2_. Lymphocytes from a healthy donor were prepared from peripheral blood according to a previously described protocol [22]. Cells were frozen in AIM V Medium (Gibco) containing 45% human-plasma-derived serum + 10% DMSO and stored at -80°C. Frozen cells were thawed in a 37°C heated water bath, and recovered in AIM V Medium at 37°C for 30 min before use. Cells in suspension were counted and their diameter was measured using a Countess Automated Cell Counter (Invitrogen, Carlsbad, CA, USA). Lymphocytes and carcinoma cells were mixed in different ratios in 100 ml of PBS and processed using the filtration device.

### Mutation analysis

Detection and quantitation of *FGFR3* mutations (p.R248C, p.S249C, p.G370C and p.Y373C) and corresponding wild type sequences were performed by droplet digital PCR (ddPCR), using the QX200 system (Bio-Rad Laboratories, Hercules, CA) and hydrolysis probe-based assays (PrimePCR ddPCR Mutation Detection Assays; Bio-Rad). The PCR mixture contained 11 μl of ddPCR droplet supermix for probes (no dUTPs), 1.1 μl of mutation primer/probe mix (FAM), 1.1 μl of wild type primer/probe mix (HEX) and 2 μl of DNA in a final volume of 22 μl. Twenty microliters of this mixture and 70 μl of droplet generation oil were transferred to different wells of a droplet generation cartridge. After formation of droplets using the droplet generator, samples were transferred to a 96-well PCR plate and subjected to amplification for 40 cycles at 94°C for 30 sec. and 55°C for 60 sec. Droplets (on average ~16,000 per reaction) were analyzed on the droplet reader, and Quantasoft software (version 1.4.0.99) was used for analyzing DNA concentrations. Cutoff settings were determined using mutation-positive and-negative control DNA samples. For urine samples processed both by filtration and sedimentation, equimolar amounts of DNA were used as template.

### DNA methylation analysis

DNA (up to 500 ng) was treated with bisulfite using the EZ DNA Methylation-Gold Kit (Zymo Research Corp, Orange, CA, USA), eluted in 20 μl of M-Elution Buffer and stored at 80°C. Quantitative analysis of methylation levels at the promoter CpG islands of *BCL2*, *CCNA1*, *EOMES*, *HOXA9*, *POU4F2*, *SALL3* and *VIM* was performed using TaqMan-based real-time PCR (MethyLight) assays, using previously described primers, probes and conditions [17]. Reactions were performed on the LightCycler 480 platform using the LightCycler 480 Probes Master Kit (Roche, Mannheim, Germany) and 1 μl of bisulfite-treated DNA per reaction. *In vitro* methylated DNA (IVM; CpGenomeTM Universal Methylated DNA, Chemicon/Millipore, Billerica, MA) and whole-genome amplified DNA served as positive and negative controls for methylation, respectively. Methylation levels were calculated as percent methylated reference (PMR; Ref. [23]) by normalizing marker-specific reaction values to *ALUC4* values relative to the same values for fully methylated control (IVM). Samples with a concentration below the equivalent of 0.25 ng/μl non bisulfite-treated DNA were excluded. For each marker, the cut-off value above which a sample was considered positive was determined by analysis of DNA from filtered urine from 11 healthy controls [17]. Cut-off PMR values for *HOXA9*, *POU4F2*, *SALL3* and *VIM* were 3, 2, 0.5 and 2, respectively, while no background amplification was observed for *BCL2*, *CCNA1* and *EOMES*.

## Results

### Outline of the filtration device

The prototype of the filtration device and its individual components are shown in Fig 1. The entire device assembly comprises 1) a filtration unit for filtering a biological fluid sample, 2) a removable filter cartridge containing a filter suitable for capturing relevant material, and 3) a storage unit for preparing and/or preserving the filter content. The filtration unit has a collection chamber, a waste reservoir, and a filter support platform housing the removable filter cartridge (Fig 1A). These three parts are connected to permit passage of a fluid from the collection chamber into the waste reservoir through the filter of the filter cartridge (Fig 1B). The collection chamber consists of a cylindrical bag surrounded by a helical spring, which can be manually compressed to generate a pressure that forces the fluid through the filter (Fig 1C). The filter support platform contains a valve that is released at high pressure if the filter becomes clogged by material, allowing direct passage of the fluid from the collection chamber into the waste reservoir. The waste reservoir contains absorbing material to permit disposal of device and fluid waste. The prototype of this device was designed with a maximum fluid capacity of 500 ml to accommodate and process entire urine voids.

After filtration, the filter cartridge with filter can be removed from the filtration unit and inserted into the storage unit (Fig 1D). The lid of the storage unit contains an appropriate treatment or storage solution that is released onto the filter when the lid is fitted. When engaged with filter cartridge and lid, the storage unit forms a sealed assembly that can be easily and safely transported (Fig 1E). Once received by an analyst, the captured biological material can be readily retrieved from the storage unit by removal of the lid.

### Evaluation of device performance

**Fig 2:**
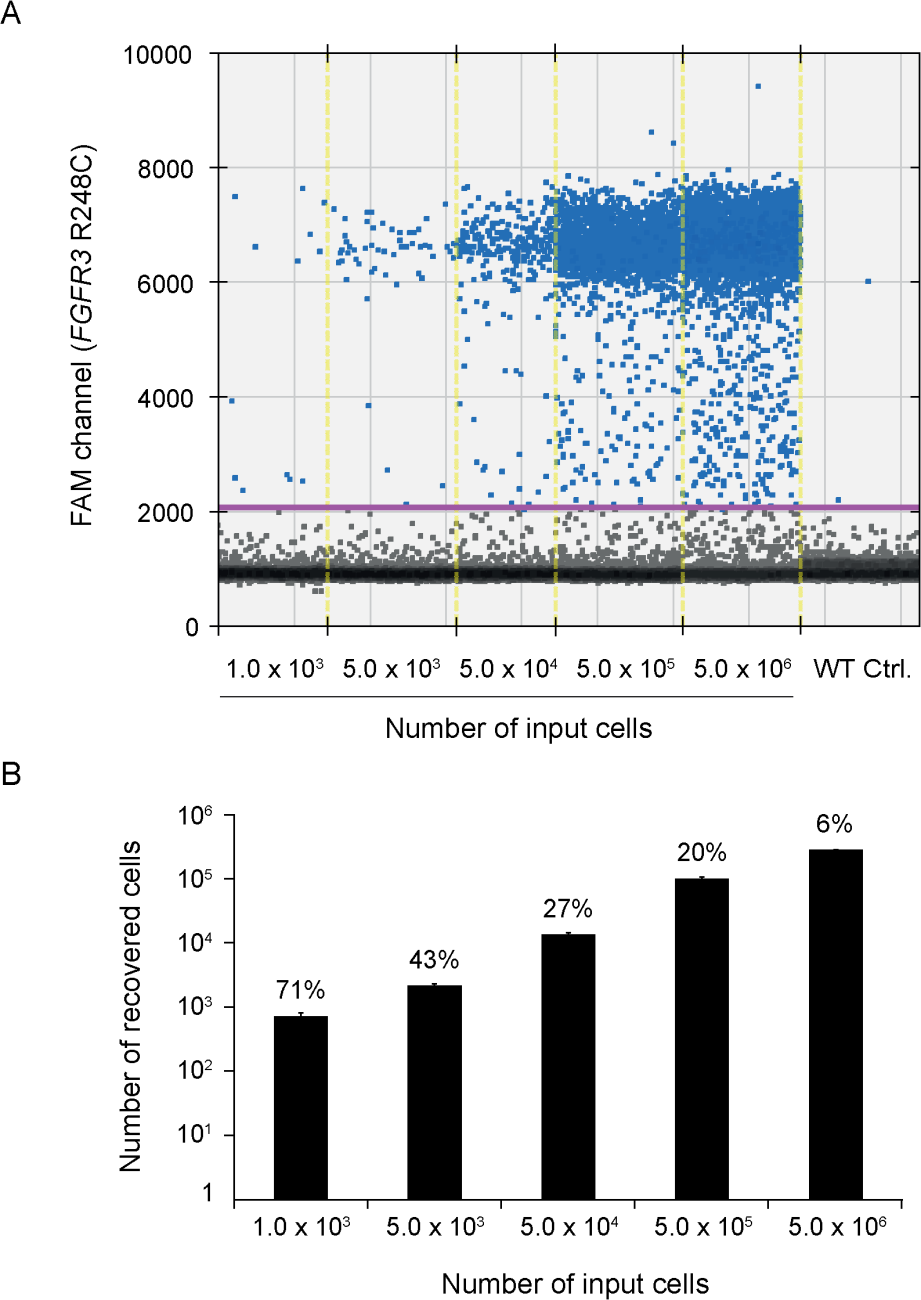
Capture of tumor cells from fluid by filtration. PBS (100 ml) containing between 1,000 and 5 × 10^6^ 639V cells was processed using a device mounted with an 8-μm pore size polycarbonate membrane filter. DNA was extracted from the filters and tested for mutant *FGFR3* (p.R248C) molecules using ddPCR. DNA from normal peripheral blood lymphocytes was used as a control for wild type *FGFR3*. A, ddPCR fluorescence amplitude plot. Vertical lines represent manually set cutoff settings. The results shown are from one of two independent experiments. B, Bar chart showing the number of recovered cells (calculated on the basis of mutant *FGFR3* molecules) relative to number of input cells. Total counts of mutant molecules were calculated on the basis of three independent ddPCR tests, each measuring mutant molecules in 4% of the total DNA sample. Data of triplicate measurements (each on 4% of total DNA) from one experiment are represented as means ± SD. Percentages above bars represent the number of recovered cells relative to the number of input cells.

As a model system to evaluate the ability of the device to capture and enumerate tumor cells from fluid samples, we used the 639V urothelial carcinoma cell line, which has a point mutation (p.R248C) in the gene encoding fibroblast growth factor receptor 3 (*FGFR3*) with loss of the corresponding wild type allele (S1 Fig). In the first set of experiments, 100 ml of PBS containing between 1,000 and 5 × 10^6^ 639V cells (diameter, 11–18 μm) were added to the collection chamber of the device and forced through a polycarbonate membrane filter with a pore size of 8 μm. To quantitate the number of cells captured on the filter, we extracted total DNA and determined the number of mutant *FGFR3* molecules using a droplet digital PCR (ddPCR) assay. In this setting, one positive event is equivalent to one cell. Positive signals were reproducibly obtained for all samples when 2 μl (4%) of the extracted DNA was used as template for ddPCR (Fig 2A). Notably, for the lowest concentration of cells (1,000 in 100 ml), the recovery was ~70% (Fig 2B). At higher concentrations of cells, there was a decrease in recovery rate, down to ~5% at 5 × 10^6^ cells/100 ml. This lower recovery was expected as saturation of the filter will cause release of the pressure valve and a direct flow of the remaining fluid and its cellular content into the waste reservoir. This initial testing suggested that the filtration device can be used to effectively capture bladder cancer cells from a fluid, and that the recovery rate is particularly high at low concentrations of cells where the capacity of the filter has not yet been reached.

**Fig 3:**
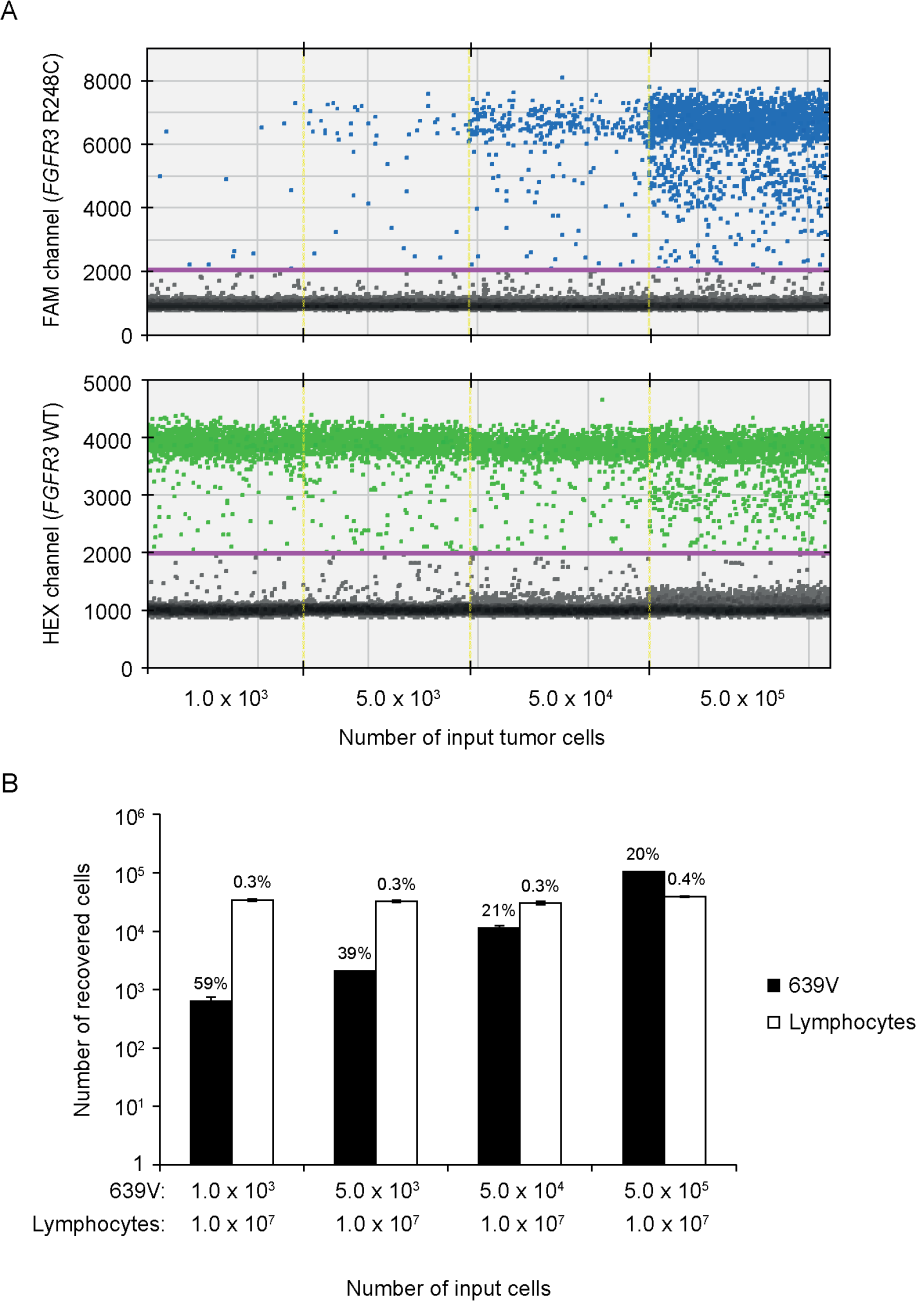
Filtration-based enrichment of tumor cells on a background of normal lymphocytes. Between 1,000 and 50,000 639V cells were spiked into 10^7^ normal lymphocytes in 100 ml of PBS, and the suspension was processed using the filtration device. DNA was extracted from the filters and tested for mutant (p.R248C; mut) and wild type (WT) *FGFR3* using ddPCR. A, ddPCR fluorescence amplitude plots of *FGFR3* R248C-FAM probe fluorescence signal (blue, upper panel) and *FGFR3* WT-HEX probe fluorescence signal (green, lower panel). Vertical lines represent manually set cutoff settings. The results shown are from one of two independent experiments. B, Bar chart showing the number of recovered cells (calculated on the basis of mutant and WT *FGFR3* molecules) relative to number of input tumor cells. Total counts of *FGFR3* molecules were calculated on the basis of three independent ddPCR tests, each using 4% of total DNA as template, and are represented as means ± SD. Percentages above the bars represent the number of recovered cells relative to the number of input cells.

To test the ability of the filtration device to increase the concentration of bladder cancer cells present in a background of smaller-sized normal cells, we spiked between 1,000 and 50,000 639V cells into 100 ml of PBS containing 10^7^ normal purified cultured human lymphocytes (diameter, 7–8 μm) and processed the suspension using the filtration device. Analysis of DNA extracted from filters by ddPCR showed signals for both mutant (p.R248C) and wild type *FGFR3* (Fig 3A). Most important, the recovery rate of mutant DNA was similar to that achieved with pure solutions of 639V cells (Fig 3B). Although the processing of samples by filtration eliminated the majority of lymphocytes (>99%), there was a consistent background of wild type *FGFR3* alleles (Fig 3), which may be derived from residual monocytes (diameter, 15–25 μm) present in the lymphocyte preparations.

To evaluate the variability of results, we filtrated 100 ml of PBS containing 500, 1,000 or 5,000 97–7 cells (diameter, 18–25 μm), each with 3–5 data points from three independent experiments. DNA was isolated and the number of mutant *FGFR3* (p.S249C) molecules was quantitated using ddPCR. In this model, the intra-sample variability was generally low and decreased with larger cell number (S2 Fig).

### Detection of bladder cancer in urine specimens

We next evaluated the performance of the device by processing urine collected from 59 patients immediately before TURB. All patients had showed evidence of bladder tumor when first examined by fluorescence-guided flexible cystoscopy. On the basis of histopathological evaluation of the biopsies, 33 of the patients were diagnosed with bladder tumors, while the remaining 26 patients had benign lesions or normal bladder epithelium. Tables 1 and 2 show the demographic and pathology data for these patients. The majority of tumors were noninvasive (stage Ta; 73%), one was a papillary urothelial neoplasm of low malignant potential (PUNLMP), two were classified as tumor in situ (Tis), four were stage T1 and two were stage T2. Twenty tumors (61%) were low grade.

**Table 1:** Demographic characteristics and molecular markers in urinary cells from patients with transitional cell bladder tumor in TURBT specimens.

			Molecular markers							
Patient ID	Sex/Age	Pathology	*FGFR3*	*BCL2*	*CCNA1*	*EOMES*	*HOXA9*	*POU4F2*	*SALL3*	*VIM*
58	F/68	High grade T2	Y373C	M	M	M	M	U	M	M
59	M/64	High grade Ta	S249C, Y373C	U	M	U	U	U	U	U
60	M/78	Low grade Ta	S249C	U	U	U	M	M	U	M
61	F/78	High grade Ta	S249C	U	M	U	M	U	U	M
63	F/70	PUNLMP	WT	U	U	U	U	U	U	U
64	M/62	Low grade Ta	WT	U	U	U	U	U	U	U
65*	M/66	Low grade Ta	NA	NA	NA	NA	NA	NA	NA	NA
67	M/74	High grade Ta	Y373C	M	M	U	M	U	M	M
69	M/72	Tis	Y373C	M	M	U	M	M	M	M
71	F/71	Low grade Ta	WT	U	M	U	M	U	U	U
72	M/67	Low grade Ta	WT	M	M	U	U	M	M	M
74	M/67	Low grade Ta	S249C	U	M	M	M	U	U	U
75	F/67	High grade Ta	WT	M	M	M	U	U	M	M
76	F/74	Low grade Ta	Y373C	M	U	M	M	M	M	M
77	M/74	Tis	WT	M	M	U	M	M	M	M
78	M/81	Low grade Ta	WT	U	U	U	U	U	U	M
79	M/65	High grade T2	Y373C	M	M	U	M	M	M	M
80	F/78	High grade Ta	Y373C	M	M	M	M	M	M	M
81	F/72	Low grade Ta	R248C	M	U	U	M	U	U	M
83*	M/53	Low grade Ta	NA	NA	NA	NA	NA	NA	NA	NA
104	F/66	Low grade Ta	WT	U	U	M	U	U	U	M
106**	M/58	Low grade Ta	S249C	M	U	U	M	U	U	M
107**	M/65	High grade T1b	S249C	M	U	U	M	M	M	M
110**	M/68	High grade T1b	Y373C	M	M	U	M	M	M	M
112	M/72	High grade T1a	WT	M	M	U	M	M	M	M
113	F/73	Low grade Ta	WT	M	U	M	M	U	U	M
119**	M/64	Low grade Ta	Y373C	U	U	U	M	U	U	M
120**	F/70	Low grade Ta	S249C	M	M	U	M	M	U	M
121**	M/79	High grade T1a	S249C	M	M	M	U	M	M	M
123	M/84	Low grade Ta	WT	M	U	U	M	M	M	M
124	M/71	Low grade Ta	WT	U	U	U	M	U	U	U
126**	M/72	Low grade Ta	S249C	U	U	U	M	U	U	M
127**	M/72	Low grade Ta	Y373C	U	U	M	M	U	U	M

*, Samples not analyzed (NA) due to insufficient DNA.

**, Samples analyzed for *FGFR3* mutations by both sedimentation and filtration.

PUNLMP, papillary urothelial neoplasm of low malignant potential. Tis, tumor in situ. WT, wild type. M, hypermethylated. U, unmethylated.

† Percentages in parentheses indicate frequencies of markers in previous studies of bladder cancer (see text for references).

**Table 2:** Demographic characteristics and molecular markers in urinary cells from patients with benign bladder histology.

				Molecular markers							
Patient ID	Sex/Age	Pathology	First visit/Follow-up (initial diagnosis)	*FGFR3*	*BCL2*	*CCNA1*	*EOMES*	*HOXA9*	*POU4F2*	*SALL3*	*VIM*
62	M/71	Inflammation	First visit	WT	U	U	U	U	U	U	U
66	M/64	Normal bladder	Follow-up (LG, Ta)	WT	U	M	U	U	U	U	U
68	M/83	Normal bladder	Follow-up (LG, Ta)	WT	M	M	U	M	U	U	U
70	K/80	Glandular metaplasia	First visit	WT	U	U	U	U	U	U	U
82	M/77	Normal bladder	First visit	WT	U	U	U	U	U	U	M
84	M/71	Normal bladder	Follow-up (Tis)	WT	U	M	U	M	U	U	U
85	M/74	Normal bladder	First visit	WT	U	U	U	U	U	U	U
86	M/75	Inflammation	Follow-up (LG, Ta + Tis)	WT	U	U	U	U	U	U	U
87	K/41	Inflammation	First visit	WT	U	U	U	U	U	M	U
90	M/74	Chronic inflammation	First visit	WT	U	U	U	U	M	M	U
91	K/74	Chronic inflammation	Follow-up (T1 + Tis)	WT	U	U	U	U	U	U	U
93	M/50	Chronic inflammation	First visit	WT	U	U	U	U	U	U	U
100	M/59	Inverted papilloma	First visit	WT	U	U	U	U	U	U	U
101	K/53	Normal bladder	First visit	WT	U	U	U	U	U	U	U
102	M/49	Normal bladder	First visit	WT	M	U	U	U	M	U	U
103	K/78	Normal bladder	First visit	WT	M	M	M	U	M	M	U
105	M/46	Inflammation	First visit	WT	U	U	U	U	U	M	U
108	M/68	Chronic inflammation	Follow-up (T1 + Tis)	WT	U	U	U	U	U	U	U
109	M/42	Inflammation	First visit	WT	U	U	U	U	U	U	U
114	M/71	Inflammation	Follow-up (LG, Ta)	WT	U	M	U	U	M	M	M
*116	M/84	Inflammation	Follow-up (LG, Ta)	S249C	M	M	U	M	U	M	M
117	K/74	Inflammation	Follow-up (LG, Ta)	WT	U	U	U	U	M	U	M
118	K/49	Normal bladder	First visit	WT	U	U	U	U	U	U	U
122	M/74	Inflammation	Follow-up (HG, Ta + Tis)	WT	U	U	U	U	U	U	U
125	M/45	Inflammation	Follow-up (LG, Ta + Tis)	WT	U	U	U	U	U	M	U
129	M/74	Inflammation	Follow-up (Tis)	WT	U	U	U	U	U	U	U

*, Patient diagnosed with a low-grade Ta tumor 6 months after a negative TURB result.

LG, low grade. HG, high grade. Tis, tumor in situ. WT, wild type. M, hypermethylated. U, unmethylated.

**Fig 4:**
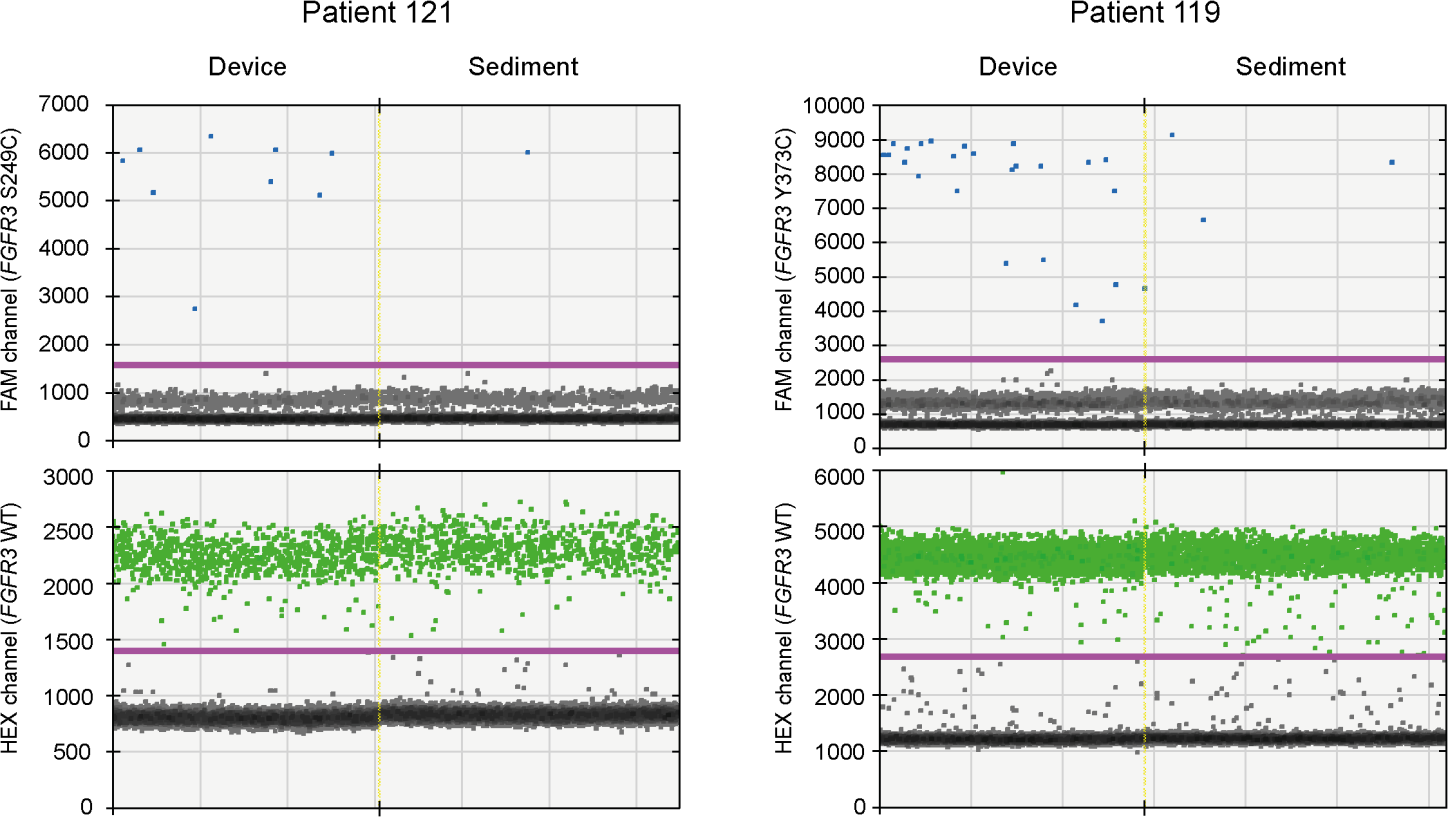
Enrichment of bladder tumor cells from urine. Urine samples from patients with bladder tumors were divided into two fractions and processed by device filtration and sedimentation, respectively. Equimolar amounts of DNA from filters and sediments were tested for *FGFR3* mutations using ddPCR.

In order to test whether filtration could increase the ratio of normal-to-tumor cells, we first tested 13 urine samples in a split-sample setup, where 25 ml of each sample were sedimented by centrifugation, and the remainder was processed by filtration. DNA isolated from all filter and sediment samples were screened for four common *FGFR3* mutations (p.R248C, p.S249C, p.G370C and p.Y373C) using ddPCR. Eight of the samples (58%) were positive for one of these mutations (Table 1). Quantitative analysis using equimolar amounts of total DNA from filters and sediments showed that the ratio of mutant-to-wild type DNA was higher in the filtered samples than in the corresponding sediments (Table 3). Most important, the greatest enrichments (6.5 and 8.0 times, respectively) were achieved for the two samples representing the lowest mutant-to-wild type ratios (Fig 4).

**Table 3:** Fractions of mutant (Mut) and wild type (WT) *FGFR3* in urinary cells from paired samples processed by device filtration and sedimentation, respectively.

		Mut/WT ± SE		
Patient ID	*FGFR3* mutation	Device	Sediment	Device/Sediment
106	S249C	0.848 ± 0.047	0.812 ± 0.014	1.05
107	S249C	0.729 ± 0.015	0.396 ± 0.004	1.84
110	Y373C	0.182 ± 0.001	0.096 ± 0.003	1.89
119	Y373C	0.008 ± 0.002	0.001 ± 0.000	7.92
120	S249C	0.041 ± 0.002	0.034 ± 0.002	1.19
121	S249C	0.006 ± 0.001	0.001 ± 0.000	6.47
126	S249C	0.022 ± 0.005	0.020 ± 0.004	1.09
127	Y373C	0.011 ± 0.001	0.004 ± 0.000	2.82

All remaining urine samples were processed by filtration alone, and DNA was extracted and examined for *FGFR3* mutations using ddPCR and seven DNA-methylation markers (*BCL2*, *CCNA1*, *EOMES*, *HOXA9*, *POU4F2*, *SALL3* and *VIM*) using MethyLight assays. Several studies of bladder cancer have reported high sensitivities (>50%) and specificities (>90%) for these markers in bladder tumors [7,8,24], and as a panel they provided a sensitivity of 94% [17].

The median DNA yield from urine samples from the 33 patients with bladder tumors was 138 ng (range 6.3 to 3,600 ng). In two samples from patients with low-grade Ta tumors, the amount of DNA after bisulfite treatment was insufficient to allow analysis of the entire biomarker panel. Table 1 shows the results for the remaining 31 samples. Nineteen of the samples contained *FGFR3* mutations (61%), with the majority of mutations being p.Y373C (50%) and p.S249C (45%). One sample contained p.Y373C and p.S249C. A total of 118 promoter hypermethylation events were identified in 29 samples (94%), with an average of 4.1 (range 1–7) events per sample. Consistent with previous studies [8,17], the highest sensitivity was obtained with *VIM*, which was positive in 81% of the cases. The six other markers had sensitivities ranging from 74% (*HOXA9*) to 29% (*EOMES*). The two samples negative for all mutation and methylation markers were from a patient with PUNLMP and a patient with a low-grade Ta tumor, respectively.

The median DNA yield from urine samples collected from the 26 patients with a benign histopathology was 271 ng (range 21 to 1,605 ng). A total of 30 promoter hypermethylation events were identified in 13 samples (50%), with an average of 2.3 (range 1–5) events per sample. The remaining samples were negative for all markers. The profile of positive markers in this patient group was different from that in patients with tumors, with *SALL3* (26%), *CCNA1* (22%) and *POU4F2* (19%) being the most frequent. Positive methylation markers were found in 8 out of 12 patients (67%) who had previously been treated for bladder cancer and were admitted to follow-up, and in 5 out of 14 patients (36%) who had no prior history of bladder cancer. Only one of the patients with benign bladder pathology had a *FGFR3* mutation detectable in urine. This patient was also positive for five methylation markers and was diagnosed with a low-grade Ta tumor at repeat cystoscopy 6 months after the negative TURB.

## Discussion

We have developed and tested a filtration device allowing easy and inexpensive collection and processing of diagnostic cells from urine. The device can be used by patients or health care providers to supply a sample of cells suitable for DNA analysis. The buffer in the storage unit ensures long-term stability of DNA at room temperature, providing ample time for sending the sample to a testing facility using the standard postal system. The simple procedure for providing urinary cells may greatly reduce the need for direct contact between patient and the healthcare system, and could be an attractive option in low resource settings where cystoscopy cannot be offered as a standard for bladder cancer detection. The focus in this study was on bladder cancer, but the same approach may be applied to other genitourinary cancers, such as upper urinary tract tumors [4] as well as other conditions where analysis of urinary cells has diagnostic, prognostic or therapeutic value.

In spiking experiments, we demonstrated that the filtration device was capable of capturing tumor cells while eliminating a large excess of smaller-sized cells. Furthermore, analysis of urine samples from patients with bladder cancer showed that filtration increased the ratio of tumor-to-normal cells compared with sedimentation of cells by centrifugation, particularly for samples where this ratio was low. It should be noted, however, that recovery and enrichment of cells by filtration depends on a range of variables, including cell characteristics (e.g., size and deformability), filter characteristics (e.g., pore size, number of pores, spacing between pores and filter material) [25] and filtration parameters (e.g., flow rate and pressure across the filter) [26].

Among patients who had a bladder tumor confirmed at histopathologic examination, urine DNA was positive for at least one of the tested biomarkers (*FGFR3* mutations and promoter hypermethylation events) in 94% of cases. This figure was encouraging considering that the majority of the patients in this cohort presented with small non-invasive tumors, which are generally difficult to detect in urine because they shed relatively few cells [27]. Yet, larger prospective studies are required to further evaluate the potential of this approach for detecting bladder cancer in a diagnostic setting. The high sensitivity for detection of bladder tumors achieved in this study may at least in part be ascribed to the large volumes (full voids) of urine processed. Zuiverloon et al. [14] showed that the sensitivity of DNA-based detection of bladder tumors increased proportionately by increasing the volume of urine and that the most reliable test was achieved on the basis of a 24-hour collection of urinary cells, similar to the standard procedures for collection of crude urine for diagnosis of other conditions such as porphyria and renal insufficiency. In this respect, the device described here may facilitate frequent and repeated sampling.

Nearly half of the patients subjected to TURB due to evidence of tumor by routine flexible cystoscopy were found not to harbor transitional cell tumor in the biopsy specimens. Interestingly, urine samples from half of these patients were positive for one or more DNA-methylation markers, despite these markers being negative in urine from healthy individuals. Previous studies have demonstrated promoter hypermethylation in normal-appearing urothelium from patients with bladder cancer [28], indicating an epigenetic ‘field defect’. Other studies have shown that DNA-methylation biomarkers can be detected in urine years after resection of a bladder tumor despite the lack of recurrence [29,30]. Epigenetically changed patches of urothelium phenotypically indistinguishable from normal urothelium may represent precursor lesions for bladder cancer and may explain the high recurrence rate. Therefore it is possible that patients with DNA-methylation markers detectable in urine may be at increased risk of developing bladder cancer later in life. In contrast to the high prevalence of *FGFR3* mutations in urine from patients with confirmed bladder tumor (67%), only one of the patients with benign histopathology had a *FGFR3* mutation detectable in urine. Interestingly, this patient was diagnosed with a bladder tumor half a year later, suggesting that urine DNA testing may detect some bladder tumors at an earlier stage than the TURB procedure.

The filtration device effectively captures cells from large volumes of urine and concentrates and stores them for convenient shipment and downstream testing. It, therefore, has potential utility for non-invasive testing for bladder cancer as an alternative or supplement to cystoscopy, to increase the quality of life for patients and reduce the economic burden on healthcare systems [31,32].

## Supporting Information

S1 Fig Loss of wild type *FGFR3* in 639V cells. DNA from a bladder tumor (Tumor 18), 639V cells and peripheral blood leukocytes (PBL) was tested for mutant (p.R248C) and wild type (WT) *FGFR3* using ddPCR.
(TIF)

S2 Fig Variability of results obtained with the filtration device. PBS (100 ml) containing 500, 1,000 or 5,000 97–7 cells was processed using a device mounted with an 8-μm pore size polycarbonate membrane filter. DNA was extracted from the filters and tested for mutant *FGFR3* (p.S249C) using ddPCR. Three to five data points were obtained for each cell dilution, in three independent experiments (I-III).
(TIF)
