# Supplementary figures and images for: Filtration Device for On-Site Collection, Storage and Shipment of Cells from Urine and Its Application to DNA-Based Detection of Bladder Cancer
Source: PLoS One. 2015 Jul 7;10(7):e0131889. doi: 10.1371/journal.pone.0131889 (PMC4495058; doi:10.1371/journal.pone.0131889)

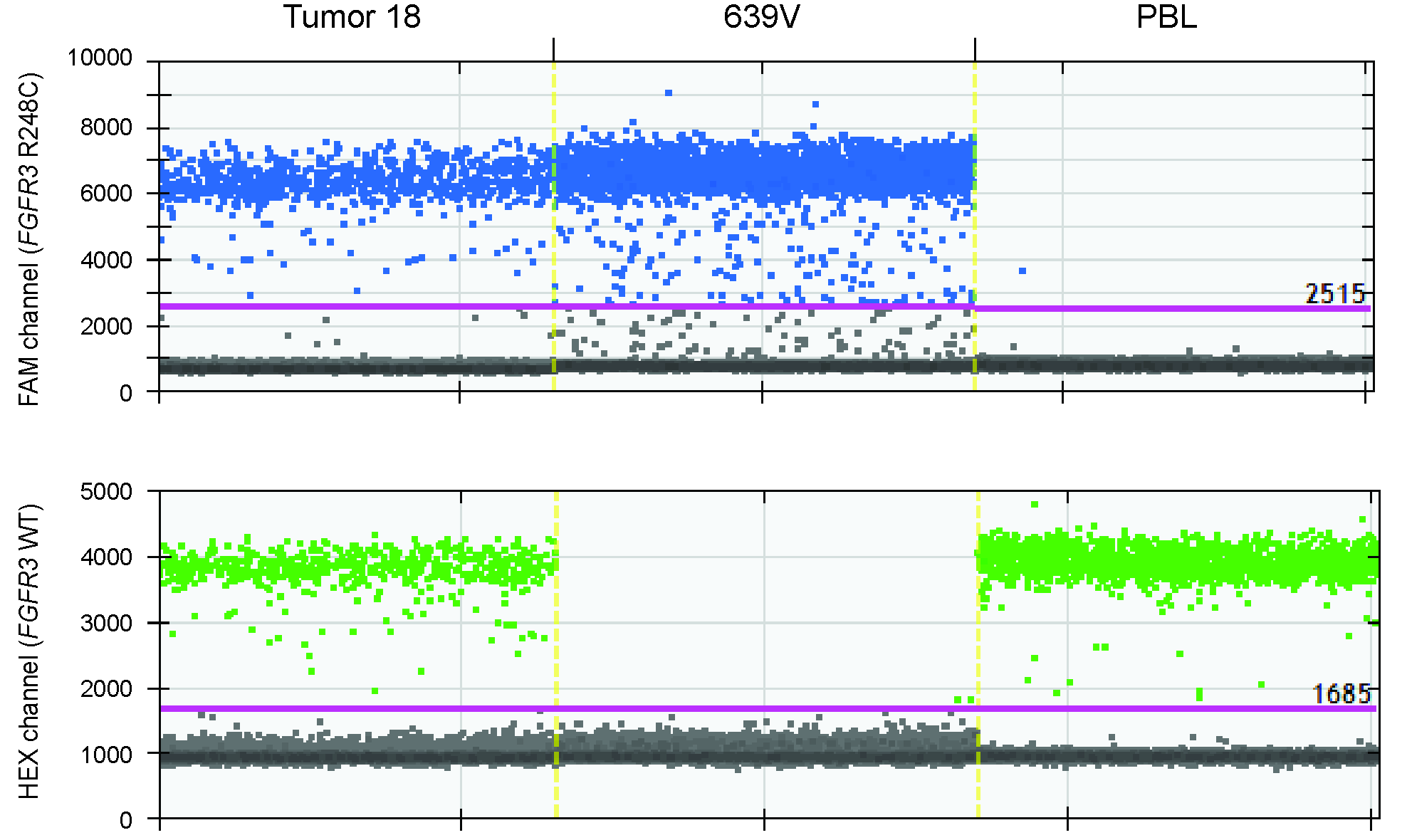

Supplement: S1 Fig — DNA from a bladder tumor (Tumor 18), 639V cells and peripheral blood leukocytes (PBL) was tested for mutant (p.R248C) and wild type (WT) FGFR3 using ddPCR. (TIF) [file pone.0131889.s001.tif]

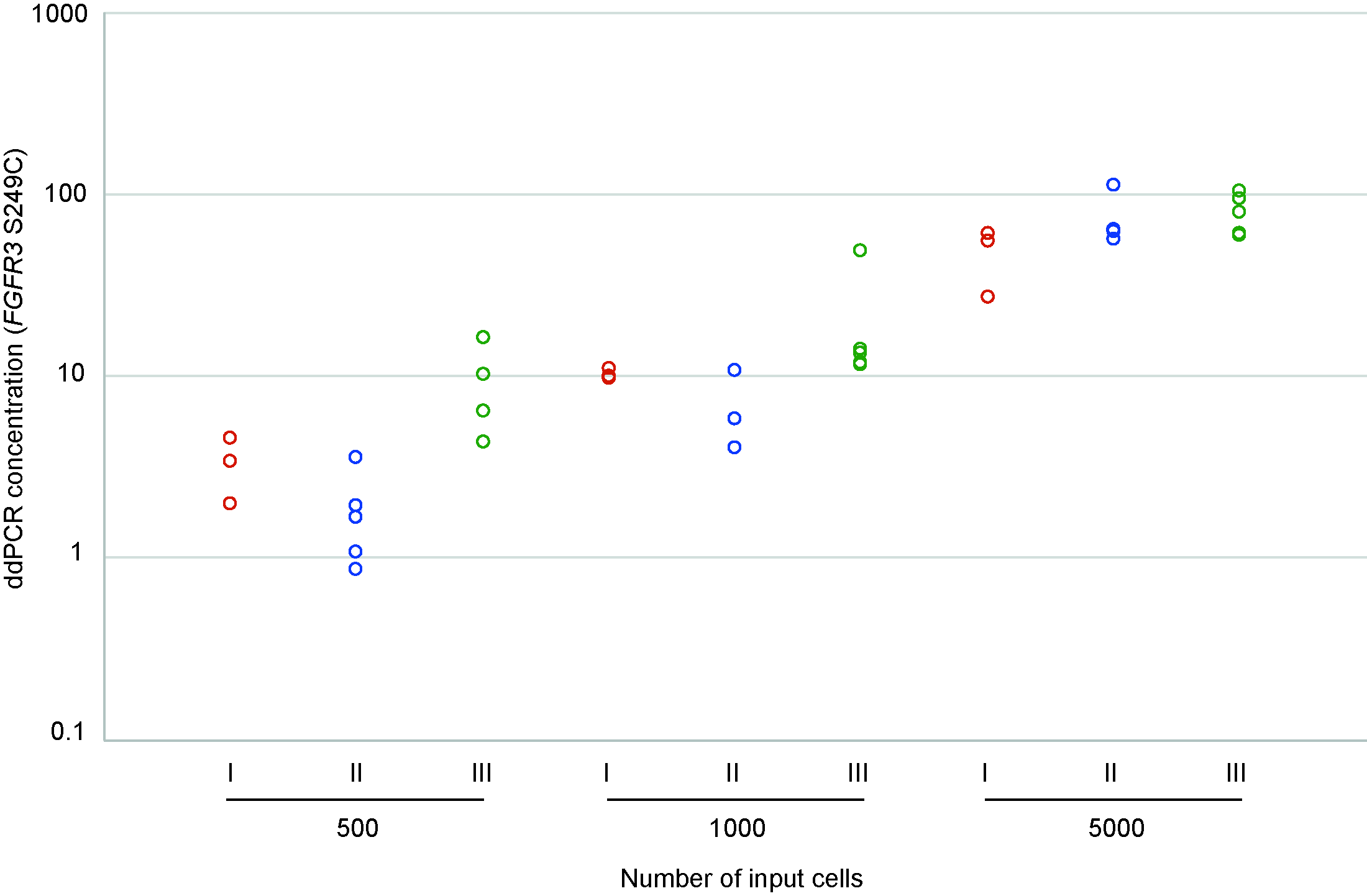

Supplement: S2 Fig — PBS (100 ml) containing 500, 1,000 or 5,000 97–7 cells was processed using a device mounted with an 8-μm pore size polycarbonate membrane filter. DNA was extracted from the filters and tested for mutant FGFR3 (p.S249C) using ddPCR. Three to five data points were obtained for each cell dilution, in three independent experiments (I-III). (TIF) [file pone.0131889.s002.tif]
